# Exploring the Untapped Potential of Neuromarketing in Online Learning: Implications and Challenges for the Higher Education Sector in Europe

**DOI:** 10.3390/bs14020080

**Published:** 2024-01-23

**Authors:** Hedda Martina Šola, Fayyaz Hussain Qureshi, Sarwar Khawaja

**Affiliations:** 1 Oxford Centre for Applied Research and Entrepreneurship (OxCARE), Oxford Business College, 65 George Street, Oxford OX1 2BQ, UK; 2 Institute for Neuromarketing, Jurja Ves III Spur no 4, 10000 Zagreb, Croatia; 3 Oxford Business College, 65 George Street, Oxford OX1 2BQ, UK; fayyaz.qureshi@oxfordbusinesscollege.ac.uk (F.H.Q.); advice@oxfordbusinesscollege.ac.uk (S.K.)

**Keywords:** neuromarketing in HE, cognitive control on Facebook design, eye tracking on website, human–robot interaction, online learning

## Abstract

This research investigates the impact of applying neuromarketing techniques to three practical examples of higher education (HE) branding: an official college website page, an official college Facebook page, and recorded online video lectures used for teaching at HE institutions. The study was conducted in three different HE institutions with a representative sample of 720 participants, with *n* = 529 used for testing the CARE college website, *n* = 59 for testing the HAZEF Facebook page, and *n* = 132 for testing the emotional response of students studying online. To assess the participants’ emotional responses, this study utilized automated facial coding through a webcam (15 Hz) and considered mood intensities. Additionally, a sentiment analysis was employed to verify the survey results and determine any discrepancies in the cognitive response. By analyzing gaze activity, movement patterns, and emotional responses, valuable insights were gained into students’ behaviors and preferences. This study recommends incorporating neuromarketing research into HE branding and online teaching to enhance students’ learning experiences. Overall, this study contributes to the understanding of human expectations and behaviors in response to online teaching and provides valuable insights for HE institutions in Europe.

## 1. Introduction

The field of neuromarketing, which combines neuroscience and marketing to understand consumer behavior and decision-making processes, has garnered significant interest in recent years. One area of application that has emerged is the use of neuromarketing techniques for online learning in higher education. This approach aims to enhance the effectiveness of online learning platforms by leveraging insights from brain and cognitive sciences. The design of learning materials is a potential area in which neuromarketing can significantly influence online learning. Traditional online learning materials often require greater engagement and interactivity to capture students’ attention and promote effective learning. Neuromarketing can provide valuable insights into the design of more engaging and effective online learning materials. By measuring neural and physiological signals such as facial coding and eye movement, neuromarketing can identify patterns and responses in learners that indicate their level of engagement, interest, and comprehension [1].

Research conducted by Smith and Seitz suggests that technology-enhanced learning has significantly contributed to higher education quality [2]. It has provided students with access to various resources and interactive learning opportunities, facilitating a more engaging and personalized learning experience. Moreover, the adoption of e-learning has expanded access to education, allowing learners from diverse backgrounds and geographical locations to pursue educational goals.

However, despite these advancements, online learning still needs to improve student engagement and retention. According to [3], neuromarketing can address these challenges by analyzing brain activity to gain insight into students’ motivation and learning preferences. By understanding neural responses to different learning stimuli, educators can design online learning experiences that are more engaging and tailored to individual learners’ needs. Furthermore, using neuromarketing in online learning can help improve memory retention and knowledge transfer.

The practice of employing neuromarketing in online learning involves measuring neural and physiological signals to gain insight into learners’ decisions, motivation, learning, and preferences [4]. Physiological tracking, which encompasses facial coding and eye movement measurements, is a frequently used method in this field. Neuromarketing is a marketing technique that aims to understand consumers’ unconscious responses; comprehend consumer preference, expectancy, motivation, and behavior prediction; and evaluate the effectiveness of advertising [5]. This interdisciplinary field combines the fields of psychology, neuroscience, and economics. The core objective of neuromarketing research is to eliminate subjectivity and ambiguity through the direct measurement of observable brain behavior, thereby enhancing behavioral predictions and comprehension of consumer behavior. Neuromarketing has also shed light on the neural variance observed in individuals in the absence of behavioral variance [6]. Technological advances have enabled neuromarketing to progress beyond traditional quantitative and qualitative research tools by focusing on consumers’ brain reactions to marketing stimuli [7] Neuromarketing research aims to connect neural activity with consumer behavior and has a wide range of applications for brands, products, packaging, advertising, and marketing for stores in determining the intention to buy, level of novelty, awareness, and emotions generated. As neuromarketing is a relatively underdeveloped discipline, theoretical research relies on neuroimaging methods to assess hypotheses, improve existing knowledge, and test the effects of marketing stimuli on consumer brains [8]. Recently, educational institutions have typically relied on conventional techniques, such as surveys and focus group discussions, to gather insights from students. However, the increasing use of technology has led to greater reliance on telemarketing and social media communication to obtain information from students and devise marketing strategies for prospective students. As a result, there has been growing interest in the application of neuromarketing techniques in the education sector. This literature review examines the use of neuromarketing in higher education, focusing on studies employing technology and neuroimaging to investigate students’ cognitive responses to various stimuli from social media and online learning environments.

### 1.1. Neuromarketing Research on Online Learning

Online learning is a self-regulated learning method that higher education has gained increased reliance on because of the COVID-19 pandemic [9]. The reasons for this shift to online learning include increased accessibility, advancements in communication technologies, and the need for institutions to remain competitive by offering flexible and varied learning platforms ([10]). Recently, the COVID-19 pandemic has necessitated the immediate suspension of in-person teaching in higher education institutions in the UK and beyond and its replacement with online learning [11,12]. Therefore, it is crucial to consider the factors that affect students’ motivation and learning performance in online classrooms. Students in higher education in Europe and the UK have become heavily reliant on online learning, and there are documented concerns about student engagement during unsupervised learning, the lack of functional group engagement, and its impact on learning capacity or mental and emotional responses to presented materials [13]. Consequently, most colleges depend on conversations with their students through interviews, surveys, and focus groups. However, researchers have documented that traditional data acquisition methods are more effective [14].

Ref. [4] conducted a study aimed at understanding the potential of online classrooms to enhance learning performance with a sample of 297 students from Oxford Business College. This study utilized stimuli-based gaze analytics to assess the impact of short and long video lectures on student motivation and learning. In the short video lecture, students’ gaze behavior was actively traced to gather data on their attention distribution, and their emotions were assessed using facial coding software. In contrast, the long video lecture assessed emotional involvement. The researchers found that students lost focus during a 90 s lecture when no accompanying visual material was used. Moreover, the results indicate that the students experienced higher levels of sadness and diminished attention when exposed to a single shareable content page for more than 5.24 min. However, when the visual material was changed and student discussions were introduced, students experienced an improvement in their mood. This study utilized several neuroscience metrics, including eye tracking, facial coding, and emotional analysis, to demonstrate the significance of using neuromarketing to enhance students’ learning performance and motivation in an online classroom. The findings of this study have practical implications for improving the learning experience and motivation of lecturers, teachers, and students in a virtual learning environment. This evidence could be leveraged to enhance online learning by boosting students’ engagement when inattentive moods are recognized.

The study of learning capacity in neuromarketing has advanced significantly using eye tracking, as demonstrated by various researchers. For instance, ref. [15] found that the integration of eye tracking and content tracking technologies in an adaptive e-learning platform allows for the delivery of relevant, accurate, and reliable information to students, tailored to their level of knowledge and behavior in real-time. Additionally, ref. [16] provided evidence for the value of eye tracking technologies in higher education, as they were found to promote strategic processing and enhance integrated image and text analysis and learning.

### 1.2. Neuromarketing Research on Social Media and Education

The concepts of user engagement and participation have become central to neuromarketing and transcending transactional contexts. In a study conducted by [17], the researchers examined the influence of content characteristics communicated by a company on Facebook on user behavior. Focusing on the medium’s form, content type, publication time, number of likes, comments, shared actions, and interaction period on the brand page, the study found that entertainment content was the most influential, whereas posts with data related to the brand increased engagement through likes and comments. Additionally, photos were the most attractive form of publication medium, and the number of comments was higher on weekdays.

Similarly, ref. [18] analyzed the impact of cultural differences on social networks and users’ commitment, loyalty, and brand recommendations. The researchers documented videos as influential in improving the number of likes, comments, and shares. The number of comments was reported to be associated with video content, and posts by the brand remained at the top of the page for an extended time.

In a study conducted by [19], the marketing techniques utilized by leading global brands on Facebook were investigated to determine the qualitative influences of messages that are most likely to elicit a consumer response. The findings of this study are consistent with previous research, indicating that images are more effective in attracting consumer responses than text-based content and, in many cases, are associated with increased engagement compared to video content. The study also found that increased content publication on social network pages was associated with elevated levels of interaction, which may have implications for fostering long-term customer–brand relationships. In 2019, ref. [2] conducted research on the rectification of neuroscience myths through Facebook, reporting that readers were more likely to positively evaluate articles that were consistent with their preexisting beliefs. In contrast, ref. [20] found that posts on Facebook, Instagram, and Snapchat were primarily used to share positive emotions, whereas Twitter and Messenger were primarily used to share negative emotions.

The advantages of neuromarketing research in the social media realm have been demonstrated in the higher education sector. The utilization of social media in academia has significantly increased, as indicated by a study revealing that almost 66% of the surveyed academics employed Twitter for information retrieval, event organization, status updates, and networking [21]. Despite this, none of the academics reported using Twitter to support assignments or engage in structured class debates. On the other hand, other studies have shown that certain academics have begun incorporating social media into their classes. A recent investigation revealed that more than 75% of students utilized social media in their professional endeavors; however, only 40% reported using it in the classroom. The primary reasons for this disparity were concerns regarding privacy and the preservation of academic integrity. Approximately half of the academics in this study concurred that social media can be beneficial in fostering collaborative learning [22].

Recent scholarly investigations conducted across European universities have disclosed the utilization of Twitter as an instrument to involve students in course materials and facilitate collaboration among peers [23,24]. Specific examples of Twitter’s applications include the establishment of a communication channel for students to submit inquiries, obtain assistance with academic tasks, and receive timely reminders about class activities. Twitter was employed to facilitate interactions between students and professors, fostering a more connected and cohesive learning environment. Research on Twitter-based assignments has revealed that activities such as posing questions, responding to statements derived from course readings, posting reactions to peers’ comments, and discussing project-related experiences can significantly contribute to enhancing students’ engagement and academic performance. Ref. [24] found that first-year students who incorporated Twitter into their seminar courses demonstrated higher levels of engagement than students in a control group who did not use Twitter. Moreover, the students who used Twitter exhibited significantly higher cumulative Grade Point Averages (GPAs) than their non-Twitter counterparts. Another study conducted by [24] revealed that although students were given the option to use Twitter, there were no significant differences in GPAs or engagement levels between students who chose to use Twitter and those who did not. Collectively, these studies underscore the potential benefits of employing neuromarketing research in the realm of social media in higher education. They also emphasized the importance of providing clear guidelines on how to utilize social media platforms, such as Twitter, for fostering interactions with professors and collaborating with peers. Incorporating social media into an educational curriculum in a purposeful and strategic manner can yield positive outcomes when implemented effectively.

### 1.3. Levying Neuromarketing Research to Higher Education 

The educational process involves students’ active engagement in the intellectual, emotional, and physical aspects of learning through various methods and techniques. Neuromarketing research in education can offer continuous improvements by gathering feedback from students to meet their requirements.

Several concerns have been raised regarding the effectiveness of higher education in enhancing students’ learning outcomes and its long-term impact [25]. In response, some educators are adopting technology to transform teaching and create more interactive learning environments in both online and in-person classrooms [25]. For example, researchers have used methods such as galvanic bracelets to measure the levels of engagement through skin responses, magnetic resonance tomography to explore the relationship between brain biology and cognitive processes, and biofeedback techniques to provide direct improvements for both learners and educators [26]. These techniques have been widely applied to various educational tools, including website design, the creation of engaging educational materials and handouts, ebooks, and multimedia, which are of interest to contemporary educators. Based on the findings of Sola’s [4] study utilizing facial coding and eye tracking software, it is suggested that academics incorporate visual shareable content every 4–5 min, utilize a visual board, and introduce class discussions to maintain student attention during long lectures. This is because prolonged lectures, exceeding 5.24 min, without shareable content result in decreased attention and poor recall and information retention. Historically, research methods employed by higher education institutions have focused on describing and predicting the effectiveness of advertising campaigns aimed at influencing student minds. Understanding and modeling students’ cognitive responses using neuroimaging techniques allows institutions to gather valuable data on subconscious processes, which can be utilized to develop effective strategies.

Neuromarketing can be a valuable tool for higher education institutions, enabling them to investigate students’ cognitive processes and the corresponding changes that occur during decision making. This can facilitate a better prediction of student behavior, both inside and outside the classroom, for example, in online learning. Additionally, neuromarketing can analyze the permanence of communication engraved in cognition regarding the scientific aspects of advertising. By utilizing this tool, institutions can devise more effective, student-driven strategies. In conclusion, the insights gained from neuromarketing research on online learning and social media can be applied to the higher education sector to increase engagement in teaching activities and promote community engagement through teaching activities that leverage social media.

## 2. Material and Methods 

The current study aims to demonstrate the potential of neuromarketing methods for enhancing university branding at a subconscious level through three practical examples. These examples include the official website page, the official Facebook page, and recorded online video lectures of higher education institutions. By utilizing neuromarketing techniques, precise and profound measurements can be made to better understand student reactions and identify enrollment behavior. This approach can be particularly useful, as conventional methods for testing and predicting the effectiveness of these investments have generally failed because of consumers’ unwillingness and inability to describe their feelings when exposed to an advertisement. Although there are challenges associated with the translation of academic research into practical applications in commercial neuromarketing, such as cost and timing, their potential benefits are significant. Therefore, neuromarketing should be considered for future implementation in higher education.

In our study on all three examples which we tested, we opted to employ the advanced quantitative research and neuromarketing research platform Tobii Sticky to measure elicited emotions. This platform enabled us to conduct remote neuromarketing research, allowing us to test a broader audience without being constrained by the geographical limitations of laboratory research. Additionally, using Tobii Sticky, we were able to capture subtle emotions through automatic facial coding with the webcam (15 Hz) modality, which traditional marketing methods cannot measure. To ensure accuracy, we tested the Tobii Sticky software and found that its average gaze error in a real-world (non-lab) environment was 1.6–1.8 degrees (approximately 5% of the screen width and 7% of the screen height) on a laptop, which provides us with reliable results regardless of the low spatial resolution of the webcam.

Moreover, a sentiment analysis was performed on two instances pertaining to the website and Facebook page trials. Regrettably, we encountered technical constraints that prevented us from executing a sentiment analysis on the archived online video lecture, as discussed in Section 6.

A sentiment analysis employs text mining and computational linguistics to determine the affective nature of written materials. The aim of a sentiment analysis is to evaluate overall consumer sentiment towards neuromarketing. By examining consumer opinions on blogs and social media, researchers have been able to identify the positive aspects of neuromarketing and its impact on consumer behavior [27].

Several studies have explored sentiment analyses on social media platforms, such as Facebook and Twitter. Ref. [28] proposed a method for ranking Facebook fan pages that considers both user engagement and comment polarity, finding it to be more accurate than traditional methods. Refs. [29,30] emphasized the potential of sentiment analysis in understanding consumer attitudes and behaviors, and ref. [30] achieved 85.25% accuracy in a sentiment analysis using a natural language processing (NLP)-based pre-processed data framework [29,30]. Refs. [31,32] further enhance the accuracy of sentiment analysis by proposing new models. Ref. [31] achieved 91.2% accuracy using a morphological sentence pattern model, while [32] experimented with a feedforward neural network for a sentiment analysis of tweets. These studies collectively underscore the growing importance and potential of sentiment analysis in the field of neuromarketing [31,32]. The sentiment analysis tool assigns a score ranging from −100 to +100, where a score of −100 indicates a very negative or serious tone and +100 suggests a very positive or enthusiastic tone.

Such an examination was not undertaken in the evaluation of the online learning material utilized in the video lecture, given the technical constraints associated with analyzing the video content. The application of neuroscience techniques in marketing, commonly known as neuromarketing, has been a subject of significant ethical debate. This includes concerns regarding the potential erosion of consumer autonomy, privacy, and control [33], as well as the capacity for manipulation and violation of autonomy and privacy. Despite these qualms, some argue that the current capabilities and implementation of neuromarketing research do not present meaningful ethical issues [33]. However, there is growing interest in the incorporation of ethical principles in neuromarketing research, as evidenced by the participation of various stakeholders in the field [34]. Ultimately, the field of neuromarketing has the potential to positively impact both society and consumers. However, ethical concerns must be addressed to ensure its responsible use [35].

We adhered to ethical standards and obtained written informed consent from all subjects on a digital form prior to their participation in the study. Participation was voluntary, and no incentives were provided. All studies were conducted in accordance with the European Code of Ethics for Research, and subject data were handled in accordance with standard practices and the General Data Protection Regulation (GDPR). The Ethics Committee of the Institute for Neuromarketing approved our research and supervised the study to ensure compliance with local and international ethical guidelines, which are publicly available on the institute’s official website.

### 2.1. Methodology

#### 2.1.1. CARE Website Page

When crafting web content, it is crucial to carefully select words to ensure optimal visibility on Google and attract students. The behavior of students on college websites and the information they obtained from the content were examined using eye tracking sensors. This research revealed that the eye movements and attention of the students differed based on their academic backgrounds. Science major students outperformed non-science major students in online scientific literacy assessments [36]. Eye tracking technology has also been used to explore how viewers process web-based multimedia information, revealing that students’ eyes are more fixated on text than on illustrations [37]. Another study focused on learning objects and found that students paid more attention to headlines and illustrations, leading to better learning performance [38]. Additionally, eye tracking was used to track the eye movement process of college students while surfing websites with different levels of complexity. The results showed that task complexity could moderate the effect of website complexity on users’ visual attention and behavior [39].

##### Experiment Setup

**Figure 1:**
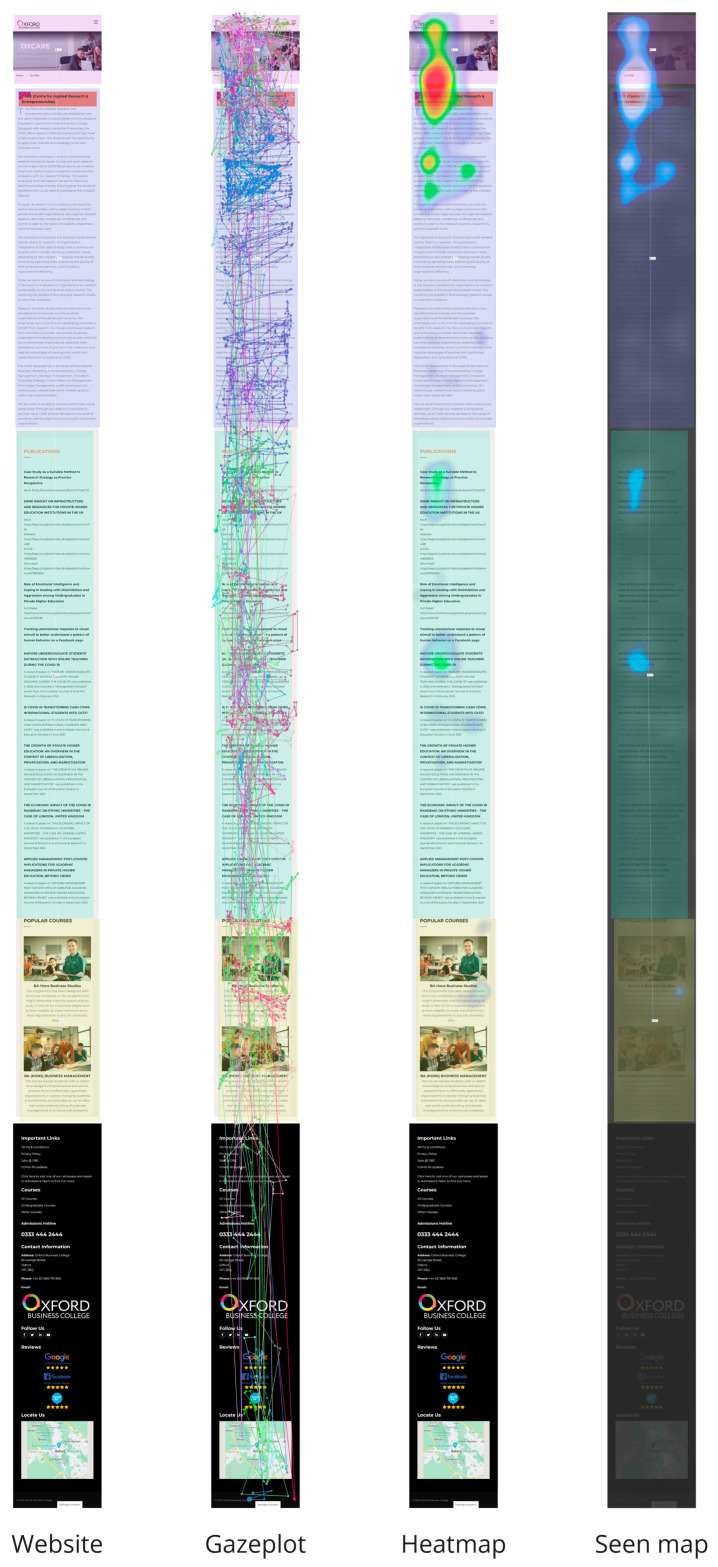
The CARE page was analyzed in this study.

In our methodology, we employed a combination of eye tracking technology and questionnaires. In our study, 529 subjects from Oxford Business College (OBC), comprising both genders and spanning the age range of 18 to 50 years with various occupations, including students, professors, and employed professionals from the UK, participated in our experiments. We aimed to analyze the recently added “CARE” page on the official OBC website, as it was the newest addition to the site, and sought to gain deeper insights into how it was perceived at a subliminal level. The content of the page was evaluated by comparing the text’s search engine optimization (SEO) position and emotional distribution through sentiment and neuromarketing analyses, including facial coding. Using G*power [40], we determined that a sample size of *n* = 35 was required to detect an effect with 90% power and a two-sided significance level of 5%. Our research sample of 529 subjects surpassed this merit in terms of the tested sample size (see Appendix A). All subjects were provided with an “HTML” link to the subpage of the Oxford Business College website and were instructed to browse the Center for Applied Research and Entrepreneurship (CARE) page on their mobile device for 90 s. Our software captured various metrics, such as heat maps, seen maps, gaze plots (Figure 1), mouse clicks, and all areas of interest (AOIs), performed for each section of the text on the website page. Heatmaps are graphical representations of complex data that utilize color to facilitate visualization and comprehension. They are commonly employed for post hoc analyses of user behavior and can reveal the most clicked area, the point at which individuals cease scrolling, general website navigation patterns, and areas of interest. Warm tones signify higher data values, while cooler tones indicate lower values. In the instance of Figure 1, the heatmap employs shades of blue, green, yellow, and red, with red being indicative of increased visibility. The seen map, also depicted in Figure 1, utilizes shades of grey, blue, and white, with lighter colors indicating higher visibility. The gaze plot, also shown in Figure 1, is a visualization that showcases individual data points connected by lines for each participant. This visualization is most useful when examining the gaze patterns of a limited number of participants, as it can become cluttered with an increasing number of individuals. The Media Only visualization, also displayed in Figure 1, presents the raw media file. It is crucial to evaluate the performance of various contexts, which is why we conducted tests on heat maps, seen maps, and gaze plots (as demonstrated in the example in Figure 1.

The participants were instructed that they could proceed to the question section only if they had read the page text earlier and were familiar with it. Statistical indicators of OBC revealed that the greatest number of visits to websites originated from mobile devices. Consequently, we conducted an experiment using mobile phones. However, owing to the reduced optical resolution and sensitivity of the software to slight changes, a larger sample size was required. The use of mobile devices in neuromarketing presents obstacles in terms of sample size and data quality. Ref. [41] found that decreasing the sample size can significantly impact research outcomes, with the threshold for comparable results increasing with the task duration. This is particularly relevant in the context of mobile device use, where the quality of image reviews can be affected by factors such as ambient light [42]. In addition, participants’ emotional reactions to the website were assessed through facial coding using their mobile phone camera to obtain insights into their emotional activity. The mood intensity measure reflects the intensity of the elicited positive and negative emotions and ranges from 0 to 1. A negative mood score indicates the intensity of negative emotions such as anger, puzzlement, fear, and sadness, while a positive mood score reflects the intensity of joy. Text from the CARE webpage was analyzed using a sentiment analysis. Finally, the participants were asked to answer three multiple-choice questions and one open-ended question. Each question had a 10 s time limit (Appendix D). Along with neuromarketing testing, we performed a sentiment analysis to understand and leverage user-generated content on a website.

Sentiment analysis is a critical tool for comprehending the opinions expressed by individuals on various websites, including social media platforms and product review sites [43]. Among the different types of sentiment analysis, aspect-based sentiment analysis (ABSA) is particularly effective for extracting sentiment features from web comments [44]. It is essential to develop an accurate website sentiment for artists’ websites, as it can heighten interest, set appropriate expectations, and ultimately lead to increased sales [45]. Researchers have also paid significant attention to analyzing online word of mouth and sentiment in online consumer reviews [46]. A novel approach that utilizes a conditional random field algorithm and support vector machine classifier has been proposed for the sentiment classification of online reviews, achieving high accuracy and eliminating the need for relying on domain dictionaries [47].

#### 2.1.2. HAZEF Facebook Page

Neuroscientific methods, such as eye tracking devices, have been utilized by researchers to track unconscious responses to visual stimuli and elucidate human behavior on Facebook pages. These devices offer valuable insights into users’ attention and emotional reactions when viewing Facebook pages [48]. Through the analysis of eye movements and fixations, researchers can explore the interrelationship between individual differences in personality, mental well-being, and the focus of users’ visual attention on Facebook [49]. Furthermore, the application of eye tracking-based brain–computer interface (BCI) systems has enabled real-time analyses of brain activity in response to visual and auditory stimuli, providing an additional understanding of human behavior [50]. By integrating these approaches, researchers can gain insights into users’ cognitive perceptions, emotional responses, and behavioral reactions to visual stimuli on Facebook pages, ultimately helping marketers to optimize content and capture user attention [51,52].

The aim of our research was to examine human behavior on Facebook and enhance the content to capture students’ attention. To achieve this objective, we employed neuromarketing methods in conjunction with a semantic analysis, which is a conventional approach in traditional marketing. This study aimed to ascertain why users follow the Facebook page of the Croatian Academic Union of the Faculty of Economics (HAZEF). Through an assessment of the present content, this investigation offers insight into the potential for attracting new followers who demonstrate sustained interest and engage with the content on an emotional level.

##### Experiment Setup

**Figure 2:**
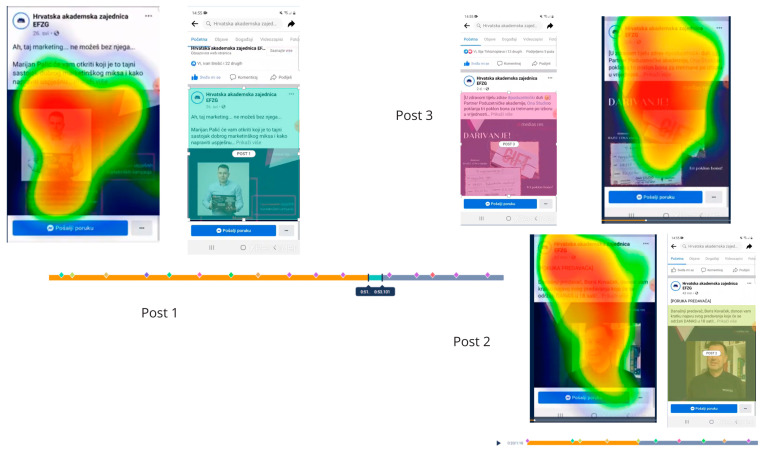
Facebook posts analyzed in this study. Post 1, Post 2, Post 3 with selected AOIs and heat maps.

A sample of 190 individuals, equally consisting of male and female participants, aged between 20 and 55 years, were chosen to participate in a neuromarketing experiment conducted on the official Facebook page of the Croatian Academic Union of the Faculty of Economics (HAZEF). Of the total number of participants, 59 successfully completed the study (the number of participants that completed the entire experiment), yielding 45 eye tracking recordings that were deemed usable (the number of participants whose gaze and/or emotion were trackable during their session, i.e., had proper lighting and did not move), with zero instances of screen-out (participants that ended their session based on a screen-out question or did not meet technical requirements) and zero partial participants (participants who started the experiment but closed the browser or had timed out before reaching the end). This study was conducted in Croatia. According to the output from G*Power, a sample size of 43 was required to detect the desired effect, with a power of 95% and a two-sided significance level of 5% (Appendix B). All subjects were presented with a pre-recorded 70.01 s video of three Facebook posts from 12 May to 13 June 2021 and were instructed to browse through them on their mobile devices as they typically would. Based on Facebook statistics, mobile devices are the primary means of visiting the HAZEF site, hence the requirement for mobile phone access in the study. The video was recorded to allow subjects to scroll through each post, from top to bottom, and vice versa, but the technical limitation of the software prevented subjects from clicking “See more” on the posts. In this study, the Facebook posts depicted in Figure 2 were analyzed. These posts included Post 1, Post 2, and Post 3, each accompanied by selected areas of interest (AOIs) and heatmaps. The attention heat map illustrates how customers direct their gaze while using the three Facebook posts, with warmer hues signifying extended periods of focus. This visualization pinpoints the content or sections that capture the viewer’s interest the most, as indicated by the concentrated red area. Conversely, green areas represent regions that require less cognitive effort. These heat maps, which provide a static representation of gaze distribution, were complemented by facial coding techniques that assessed the emotional responses of subjects based on their facial expressions. This analysis aimed to evaluate the influence of HAZEF’s Facebook page, as shown in Figure 2. The three Facebook posts displayed relevant announcements regarding HAZEF’s Entrepreneurial Academy at the Medias Res conference. Along with eye tracking testing, these posts were analyzed using a sentiment analysis tool after being translated into English, as the sentiment analysis platform conducted analyses on written English texts. The sentiment analysis was performed only on the text of the Facebook posts, while a neuromarketing analysis was conducted on both text and images.

#### 2.1.3. Online Lecture at Higher Education Institution

The integration of eye tracking technology in online lectures at higher education institutions holds great potential for enhancing learning experiences. Eye tracking enables researchers to monitor students’ reading and learning behaviors, offering valuable insights into their cognitive processing and topic interests [53]. By tracking students’ eye movements, researchers can assess their attention to various stimuli, such as the instructor’s gaze, written information on the board, and crucial sentences in the text [54,55,56]. This information can be used to design video lectures that optimize students’ learning experiences and engagement. Furthermore, eye tracking data can be combined with artificial intelligence algorithms to predict student performance and provide personalized feedback [57]. In summary, the implementation of eye tracking in online lectures presents a promising approach for enhancing the effectiveness of higher education instruction.

##### Experiment Setup

The impact of online teaching in higher education on students’ emotional responses was investigated in Zagreb, Croatia, using a sample of 132 students (both genders) studying German at the Faculty of Teacher Education at the University of Zagreb. A priori calculations were performed using G*Power to ensure that the required sample size was achieved. A sample size of *n* = 42 (*n* = 21 per device condition) was required to detect the effect with a power of 80% and a two-sided significance level of 5% (Appendix C). Students were randomly assigned to two conditions: computer (*n* = 68) and mobile phone (*n* = 64). The main objective of this study was to determine whether there is a difference in the elicited emotional responses between the two devices during online learning. The two video lectures were pre-recorded and uploaded to Tobii Sticky, with students receiving the “HTML” link to one of the video lectures according to their condition. The lecture viewed on the computer device lasted five minutes, while that viewed on the mobile phone lasted 69 s. Although a sentiment analysis could have been theoretically implemented in this study, it was not performed because of the technical restrictions of the available sentiment analysis tools. To perform a sentiment analysis using free online sentiment analysis software, videos must be publicly posted on YouTube. Unfortunately, a sentiment analysis could not be conducted on the lecture material, as it was subject to copyright and intended exclusively for students enrolled in the course. Nevertheless, even if conducted, its findings would have been limited, as they would only have provided insight into the sentiment of the lecture but not the student satisfaction score. In contrast, a neuromarketing analysis can reveal subconscious reactions to visual, auditory, and textual stimuli.

## 3. Results

### 3.1. CARE Website Page

**Figure 3:**
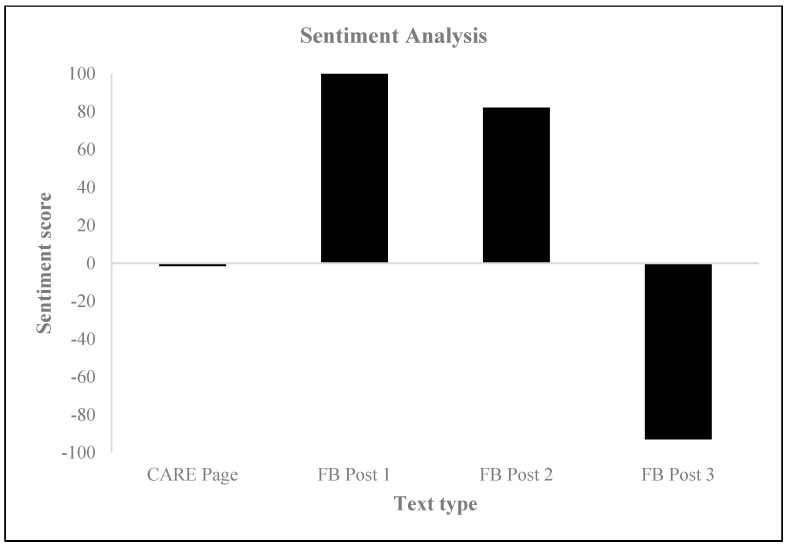
Sentiment analysis.

**Figure 4:**
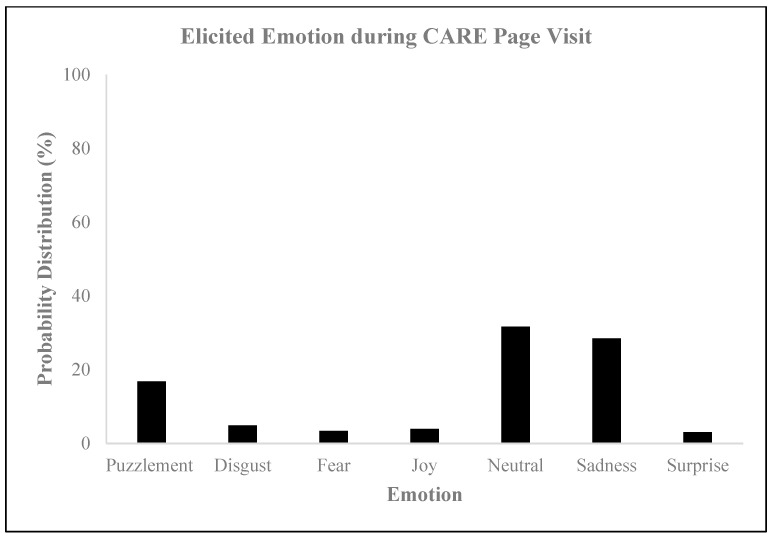
Elicited emotions during the CARE page visit.

**Figure 5:**
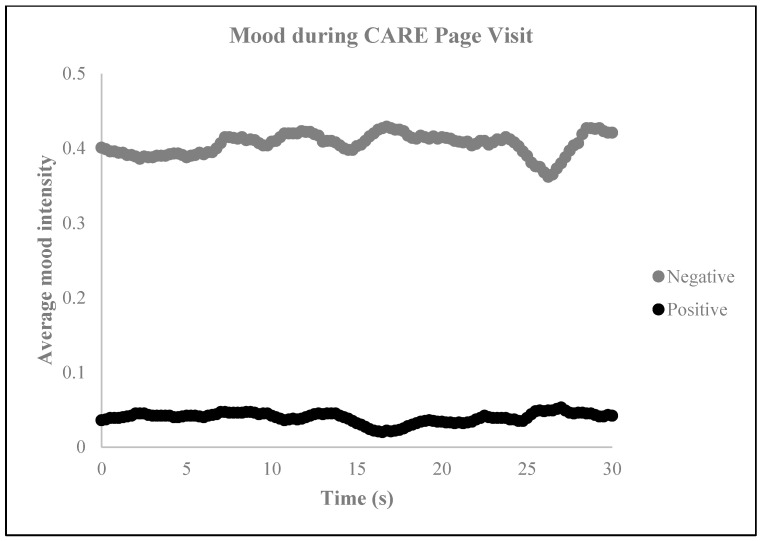
Mood intensity during the CARE page visit.

The sentiment analysis of the CARE webpage yielded a sentiment score of −1.5, indicating a predominantly negative or profound sentiment or tone in the text (Figure 3), which was substantiated by the results of the neuromarketing analysis. This analysis revealed that the subjects mostly experienced neutral and sad emotions (Figure 4) and that the general mood intensity was relatively negative (Figure 5). Furthermore, the eye tracking behavioral neurometrics, emotion analysis, and survey answers suggest that a redesign is necessary, as the information of interest for visitors is not optimally positioned on the website, and much of it goes unnoticed. It is important to note that eye tracking analyses on websites can be instrumental in capturing emotions and can be used to explore information acquisition, emotional experience, and behavioral intention on different information displays [58]. Additionally, eye tracking can be employed to offer interactive and personalized online shopping experiences on mobile smartphones [59]. The detection of emotions using eye tracking is a relatively novel approach, but it is gaining popularity in affective computing [60]. Finally, gaze direction can play a vital role in understanding and processing large volumes of image and video content, personalization in human–media interaction, visual content design, and affective analysis [61].

### 3.2. HAZEF Facebook Page

**Figure 6:**
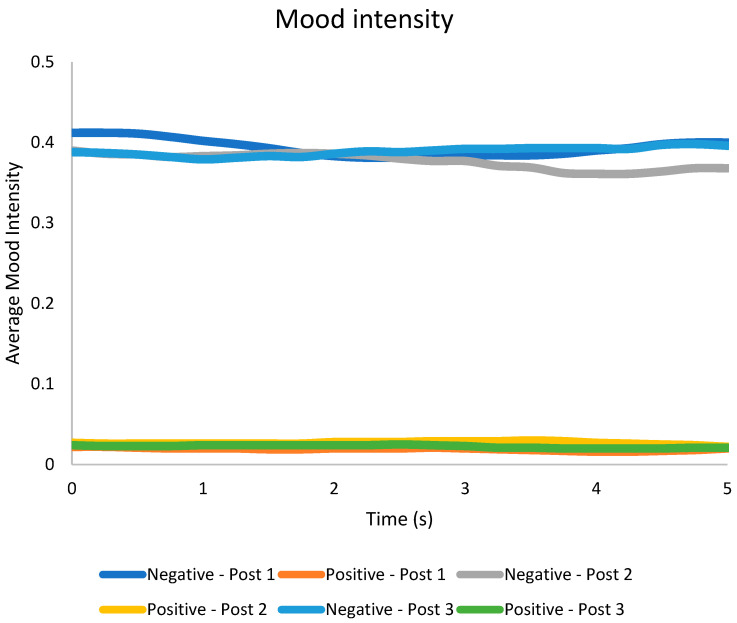
Mood intensity during Facebook page visits.

**Figure 7:**
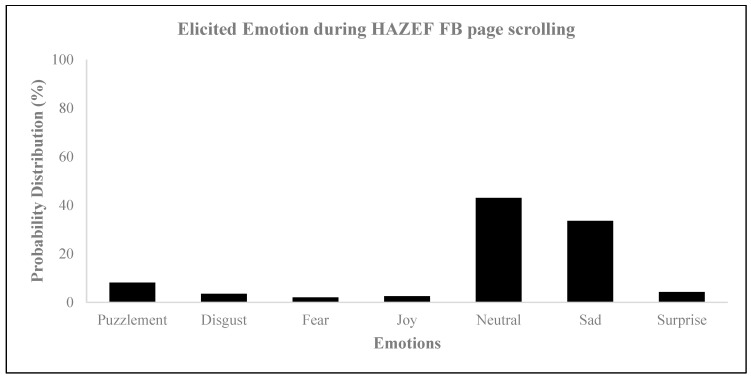
Elicited emotion during HAZEF FB page scrolling.

**Figure 8:**
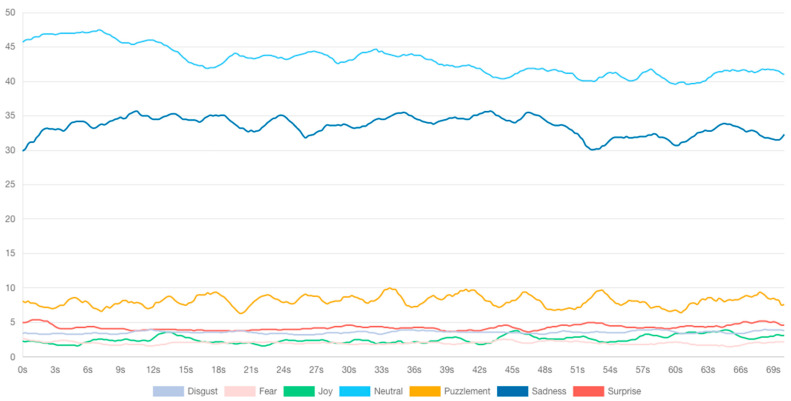
Emotional analysis of HAZEF FB page scrolling.

**Figure 9:**
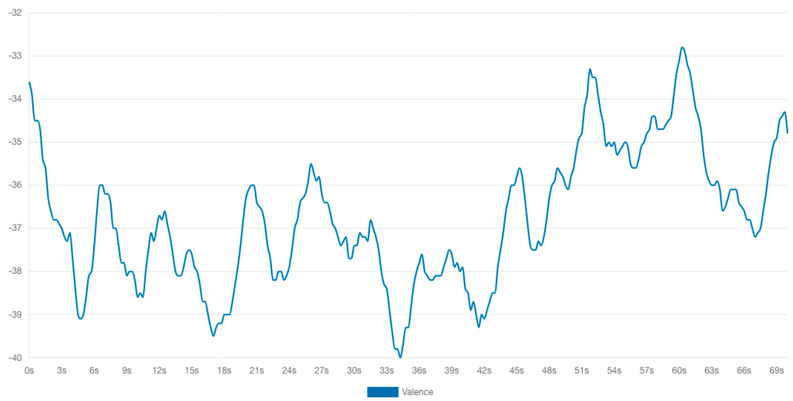
Emotional valence chart.

Upon examining the sentiment scores for the HAZEF Facebook posts, it is evident that the results were inconsistent. Specifically, Facebook Post 1 attained a total score of 100, indicating a predominantly positive and zealous sentiment. A similar overall tone was observed for Facebook Post 2, which achieved a score of 82.1. Conversely, the sentiment score for Facebook Post 3 was −93.0, suggesting a negative and solemn sentiment. Furthermore, the assessment of emotional responses through eye tracking revealed that the subjects exhibited heightened levels of negative emotions (as shown in Figure 6) and more intense feelings of neutral and sad emotions (as illustrated in Figure 7, Figure 8 and Figure 9), regardless of the type of post. Eye tracking analysis on Facebook is important for capturing emotions [50,60,62] because it allows researchers to explore the relationship between individual differences in personality, mental well-being, SNS usage, and the focus on Facebook users’ visual attention [63]. Eye tracking technology can be used as a primary sensor modality for emotion detection, alongside other methods such as EEG, facial image processing, and speech inflections [61]. The preliminary results from an eye tracking study indicate that dynamic body features, such as torso and arm movements, are attended to most often and longest, suggesting their importance in decoding emotions. Eye tracking can also be used to analyze the distribution of visual attention on Facebook pages, providing insights into users’ visual attention and preferences for different types of advertisements. Overall, eye tracking analyses on Facebook can contribute to the understanding of emotions, personalization, visual content design, and affective analysis.

### 3.3. Online Lecture at Higher Education Institution

**Figure 10:**
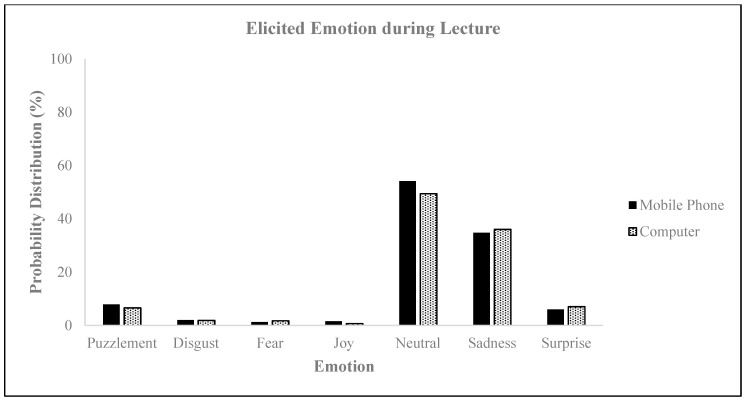
Elicited emotions during lectures.

**Figure 11:**
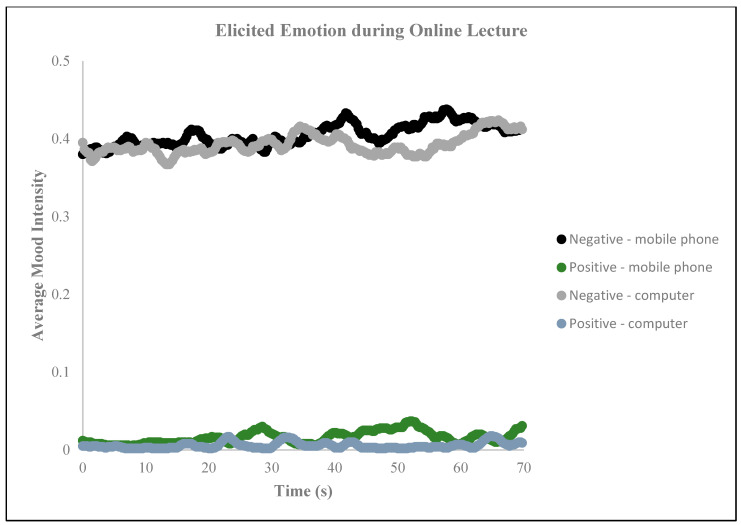
Elicited emotions during online lectures.

The results of the neuromarketing assessment conducted on the emotional reactions of the participants during the first 70 s of the lecture revealed that, regardless of the type of device used, elevated levels of neutral and sad emotions were experienced (as depicted in Figure 10), and the intensity of negative emotions was heightened (illustrated in Figure 11). The eye tracking analysis of online lectures in higher education is crucial for capturing emotions and improving the learning process [55]. By monitoring students’ eye movements, researchers can gather valuable data on their behavior and attention levels during online learning [57,64,65]. This information can be used to understand students’ content coverage, reading patterns, and attention at both the perceptual and conceptual levels [54]. Moreover, eye tracking can help identify students’ levels of processing and topic interest, allowing for personalized learning experiences that spark their interest. Furthermore, eye tracking can be used to maintain student attention and vigilance throughout an entire online lecture, not just in the first few minutes. In conclusion, eye tracking analyses of online lectures can provide insights into students’ emotions, attention, and learning behaviors, enabling the development of adaptive learning strategies and personalized feedback to enhance the learning process.

## 4. Experimental Analysis

To assess the efficacy and implications of neuromarketing techniques in European higher education, it is necessary to conduct an experimental analysis using statistical techniques. This study aims to broaden our understanding and develop measurements for branding neuromarketing methods through the examination of emotions and mood intensities experienced on social media websites. The statistical methods employed in this study included a linear regression, MANOVA, and Pearson’s correlation.

The key findings of this study are as follows:

1. A linear regression analysis was used to examine the relationship between and the effectiveness of mood intensities, specifically positive and negative moods.
2. The MANOVA provided insight into the variation in emotions, such as puzzlement, fear, disgust, neutrality, joy, sadness, and surprise, observed by the subjects.
3. Pearson’s correlation was used to analyze the strength and direction of the emotions.

### 4.1. Demographic Analysis of CARE Website Page

The survey was conducted with a sample of 529 students, professors, and professionals who were randomly selected from the Oxford Business College. Following the examination and filtering of the data, 529 questionnaires were used for the analysis. The survey respondents were diverse and had a wide range of characteristics. Among the surveyed respondents, the percentage of male participants was 40%, which was lower than that of the female respondents (60%). Many of the respondents were students, while professors and professionals comprised equal proportions. When considering the sample population, 40% fell within the age range of 18–38 years, 30% were under 28 years of age, and 30% were above 38 years of age (Table 1).

### 4.2. Linear Regression for CARE Website Page and HAZEF Facebook Page

A linear regression measures the extent to which the independent variables impact the dependent variables and, consequently, the direction of influence, whether positive or negative. The data provided pertain to the relationship between time and mood and whether it has a positive or negative effect.

The analysis of variance (ANOVA) model was instrumental in comprehending the variability in the model. The regression model revealed that the dataset exhibited 11% variability, signifying minor fluctuations in the dependent variable. The t- and *p*-values yielded substantial and statistically significant outcomes, demonstrating a correlation between the dependent and independent variables. Notably, the *p*-value was below the threshold, indicating the model’s confidence level and verifying that the model was statistically significant (Table 2).

### 4.3. Relationship and Effectiveness of Mood Intensities between the Variables

**Figure 12:**
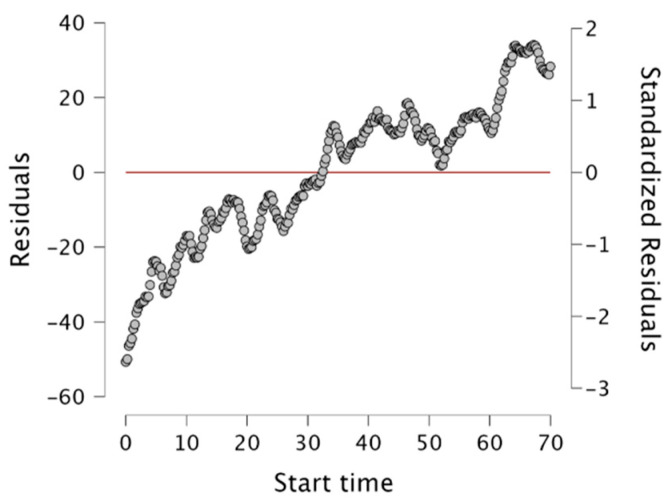
Residuals vs. dependent variable.

**Figure 13:**
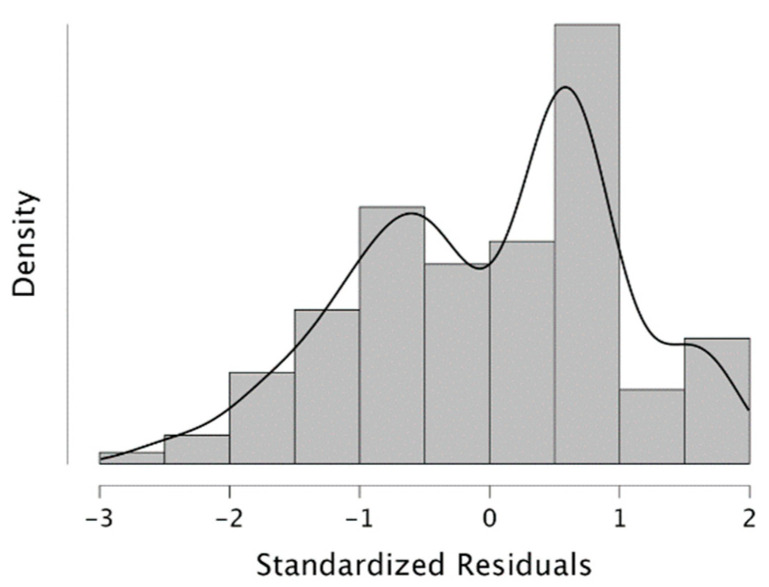
Standardized residuals histogram.

The scatter plots illustrate the linear relationship between variables in the linear regression analysis, which considers the interdependence between variables. The findings indicate that a 10% change in the online dependent variable explains 90% of the variation in the independent variable (see Figure 12). This also implies that the remaining 90% of the variability can be attributed to other factors (Figure 13).

### 4.4. MANOVA

The multivariate analysis of variance was conducted to test the significance of one or more independent variables in a set of two or more dependent variables. The dependent variable in this study was gender, with two categories: male and female. The test allowed for the simultaneous analysis of two or more samples. The normality of the data was assessed based on skewness, which was calculated to be within the acceptable range for a MANOVA. The Pillai trace statistic (1.000) indicated a significant relationship between gender and the response variables, suggesting that the volunteers who participated in the study established a significant association between gender, dependent variables, and emotions. The data showed that each volunteer had a substantial pattern of feelings and relativity (Table 3). The residuals in the multivariate analysis refer to the unexplained data. Approximately 278 degrees of freedom were associated with the residuals, which were the differences between the observed values and those predicted by the MANOVA model. The residual variability was used to measure how well the data fit into the model.

### 4.5. Assumption Checks

Box’s M-test was used to assess the homogeneity of two or more covariance matrices. The value of Box’s M was 937.883, which adhered to a chi-square distribution with 90 degrees of freedom. The resulting *p*-value was less than 0.001, indicating that a multivariate normal distribution was followed, and the variance–covariance matrices were not equivalent across the cells (Table 4).

### 4.6. Pearson Correlation 

The Pearson correlation table provides a correlation constant, its significance, and *p*-values, which indicate the strength and significance of the relationships between the variables in question. These values are of great importance in quantifying the strength and direction of linear relationships. It is worth noting that the majority of the *p*-values associated with these pairs of variables are below 0.05, indicating that their relationships are meaningful. However, it should be noted that the correlation between time and the other variables was not substantial, as indicated by the *p*-values (*p* > 0.05). The relationship between puzzlement and various emotions was also investigated. It was found to be positively correlated with sadness (r = 0.184) and negatively correlated with disgust (r = −0.094, *p* = 0.116), fear (r = −0.210, *p* < 0.001), joy (r = −0.105, *p* = 0.087), neutral emotions (r = −0.103, *p* = 0.084), and surprise (r = −0.084, *p* = 0.159). However, this relationship was not statistically significant. Disgust displayed distinct relationships with the variables, showing a significant negative correlation with fear (r = −0.125, *p* = 0.036), neutral emotions (r = −0.134, *p* < 0.001), sadness (r = −0.032, *p* = 0.596), and surprise (r = −0.002, *p*: 0.978). It also exhibited a positive correlation with joy (r = 0.195, 0 < 0.001). Fear was found to have a significant negative correlation with joy (r: −0.224, *p* < 0.001) and sadness (r: −0.101, *p*: 0.092) and a significant positive correlation with neutral emotions (r: 0.010, *p*: 0.863) and surprise (r: 0.148, 0: 0.013). Joy showed a significant positive correlation with surprise (r = 0.141, *p* = 0.018) and a significant negative correlation with neutral emotions (r = −0.493, 0 < 0.001) and sadness (r = −0.164, *p* = 0.006). Neutral emotions displayed significant positive and negative correlations with sadness (r = 0.310, *p* < 0.01) and surprise (r = −0.129, *p* = 0.031), respectively. Sadness exhibited a significant negative correlation with surprise (r: −0.633, *p* < 0.001). All these relationships were statistically significant. The directions of the correlations are represented by negative and positive signs, which also indicate whether they tend to move in the same or opposite direction when plotted in a graph (Table 5).

## 5. Conclusions

In this study, we conducted three separate neuromarketing investigations, employing eye tracking to uncover subtle emotional responses, survey questionnaires to compare subconscious shifts with cognitive reactions, and a sentiment analysis to scrutinize variations in the outcomes. Deemed worthy of merit, we decided to examine three commonly utilized domains within higher education: an official college website, an official college Facebook page, and recorded online video lectures that are fundamental to teaching at a higher education institution. For our research, we utilized a highly representative sample size of *n* =720 participants, where we used *n* = 529 to test the CARE college website, *n* = 59 to test the HAZEF Facebook page, and *n* = 132 to test the emotional response of students studying online. With the intention of gaining meritorious results, we employed three different higher educational institutions (Oxford Business College, Croatian Academic Union of the Faculty of Economics, University of Zagreb, Faculty of Teacher Education), as each institution possesses distinct branding strategies that are associated with the three measured segments. 

Our findings from the HAZEF Facebook research revealed inconsistencies in the sentiment analysis and eye tracking results. Regrettably, the sentiment analysis produced false positive outcomes, while the eye tracking analysis revealed profound negative emotions, particularly capturing sadness. This research emphasizes the significance of incorporating neuromarketing research into social media marketing research to uncover the actual behavioral patterns and emotional states of participants. Facebook eye tracking analysis can contribute to comprehending emotions, personalization, visual content design, and affective analysis. We believe that the results where participants may have paid less attention than other posts on the page are due to the color combination utilized for the visuals. The analysis of the participants’ facial expressions suggests that visiting the HAZEF Facebook page evokes emotions of neutrality and sadness, which is consistent with the muted colors that are prevalent on the page. The possible implications of the elicited negative emotions as a reflection of the conference are discussed. Research in the fields of color psychology and marketing has consistently demonstrated that color can exert a substantial influence on mood and attitude. A study conducted by [66] revealed that cool background colors tend to engender more positive attitudes and behavioral intentions, particularly in positive-mood and low-involvement conditions. Similarly, ref. [67] emphasized the importance of colors in shaping moods and feelings, with the potential to attract a larger customer base. Ref. [68] further investigated the relationship between color attributes and emotional dimensions, suggesting that the cognitive quantity of color, as well as the presentation medium, can impact emotional responses.

Our CARE website research confirms the results of the sentiment and neuromarketing analyses. By comparing these results with those of the cognitive survey testing, sadness exhibited a significant negative correlation with surprise. However, this does not provide substantial insights into why these emotions persist among visitors on college websites. Employing neuromarketing research is essential to gain deeper insights into the persistence of these emotions and what a college can modify in the future to elicit more favorable emotions from its visitors. This research has validated the utility of eye tracking technology in college website testing and highlights the limitations of traditional marketing methods when seeking to understand the visitors who access our website and their genuine feelings towards it. The analysis of eye movements on websites in higher education is essential for gaining insight into the emotions of students. This method provides valuable information on students’ behavior, preferences, and needs, which can be used to design customized e-learning environments that cater to each student’s requirements [27,37,69,70]. By tracking the gaze of students, researchers can understand how they interact with the website and what content they focus on, which can be used to intervene and support students who may struggle with attention or performance issues [71]. Moreover, the relationship between eye movements and emotional states has been found to be highly correlated, making it possible to gain insight into the psychological state of students during online learning [15]. Thus, the analysis of eye movements is critical for understanding the emotions of students in higher education and enhancing their learning experience.

Our investigation at a higher education institution, which focused on online lectures, was the sole study among the other two that we conducted because of copyright restrictions. This research was unique in that it exclusively utilized eye tracking for the semantic analysis, as detailed in Experiment Setup. Our findings are insightful and highlight the need for this type of research to be conducted more frequently in higher education. The study revealed that, regardless of the device used for online lectures (mobile or desktop), the participants experienced heightened levels of neutral and sad emotions. The findings of our study on online learning demonstrate how advanced quantitative research based on eye tracking data and facial coding can provide insight into students’ attention levels and emotional engagement during online lectures. The expansion of online learning is driven by advances in communication technologies and the need for flexibility [3,67,69]. Emotional engagement is another important factor influencing students’ online learning [72]. Facial coding was used in our study to obtain details about immediate emotional responses and general mood, and the results showed that a neuromarketing analysis can provide time-based insights into mood intensity and emotional engagement, which can inform adjustments to lectures and pinpoint areas of weakness during the teaching process.

## 6. Limitations of Study 

It is imperative to acknowledge the limitations of the sentiment analysis and the Tobii Sticky platform. Conducting research simultaneously with both computers and mobile phone devices using Tobii Sticky is not possible. Moreover, the software has a technical limitation that precludes clicking “See more.” Sentiment analysis is a valuable tool; however, it has certain limitations. It can only be applied to content published on YouTube and requires Python for prerecorded videos, which restricts its use in copyrighted or exclusive videos. Additionally, a sentiment analysis does not provide insights into student satisfaction or dissatisfaction with online learning. The use of neuromarketing in online learning offers a means of measuring student satisfaction and mood during lectures, which directly affects learning outcomes. By monitoring a student’s mood throughout a lecture, valuable feedback can be obtained to improve the lectures and identify areas of weakness. Although sentiment analysis has limitations, the statistical analysis conducted in this study using a linear regression, Pearson correlation, and MANOVA demonstrated a significant model for understanding human expectations and behaviors in response to online teaching. The incorporation of neuromarketing techniques in online learning can enhance user experience and engagement by identifying students’ subconscious preferences and cognitive load, leading to increased satisfaction, better learning outcomes, and an improved institutional reputation. The implications of incorporating neuromarketing techniques for online learning in higher education in Europe are far-reaching and significant, but more research is needed in this field.

## 7. Recommendation for Future Research 

Future research utilizing eye tracking technology in higher education should concentrate on several key areas. First, it is crucial to investigate the potential of eye tracking in studying self-regulated learning processes in university students [73]. This includes examining the judgments of learning, metacognitive monitoring, meta-comprehension, and learning strategies. Second, researchers should consider employing eye tracking to evaluate and refine the textual and visual elements of educational presentations [74]. This approach can provide valuable insights into tailoring educational content for different types of students [55]. Furthermore, eye tracking can be incorporated into lectures and classes to promote student engagement in research activities within their field of specialization. Lastly, future research on eye tracking on social media in higher education should concentrate on exploring how eye tracking can enhance self-regulated learning processes when students are learning from multimedia materials [71]. Additionally, research should examine the utilization of social media by higher education academics, including the advantages and challenges that they encounter. This can involve studying how academics use social media to disseminate research findings, advance their careers, and teach [73,75].

## Figures and Tables

**Table 1:** Demographic analysis.

Variables	Options	Percentages (%)
Gender	Male	40
	Female	60
Profession	Students	40
	Professors	30
	Professionals	30
Age	18–28	30
	28–38	40
	38–50	30

**Table 2:** Linear regression—model summary—start time.

Model Summary—Start Time									
Model	R	R^2^	Adjusted R^2^	RMSE	R^2^ Change	F Change	df1	df2	*p*
H₀	0	0	0	20.315	0		0	280	
H₁	0.32	0.103	0.099	19.28	0.103	31.885	1	279	<0.001
ANOVA									
Model		Sum of Squares	df	Mean Square	F	*p*			
H₁	Regression	11,852.201	1	11,852.201	31.885	<0.001			
	Residual	103,709.05	279	371.717					
	Total	115,561.25	280						

**Table 3:** MANOVA: Pillai.

Test Cases	df	Approx. F	Trace_Pillai_	Num df	Den df	*p*
(Intercept)	1	152,503.9	1	9	270	<0.001
Gender	2	19.017	0.774	18	542	<0.001
Residuals	278					

**Table 4:** Box’s M-test for the homogeneity of covariance matrices.

Box’s M-Test for Homogeneity of Covariance Matrices		
χ^2^	df	*p*
937.883	90	<0.001

**Table 5:** Pearson correlation constants and significance.

Variable		Time	Puzzlement	Disgust	Fear	Joy	Neutral Emotions	Sad	Surprise
1. Time	Pearson’s r	—							
	*p*-value	—							
2. Puzzlement	Pearson’s r	0.089	—						
	*p*-value	0.136	—						
3. Disgust	Pearson’s r	0.373	−0.094	—					
	*p*-value	<0.001	0.116	—					
4. Fear	Pearson’s r	−0.166	−0.21	−0.125	—				
	*p*-value	0.005	<0.001	0.036	—				
5. Joy	Pearson’s r	0.555	−0.105	0.195	−0.224	—			
	*p*-value	<0.001	0.078	<0.001	<0.001	—			
6. Neutral emotions	Pearson’s r	−0.875	−0.103	−0.314	0.01	−0.493	—		
	*p*-value	<0.001	0.084	<0.001	0.863	<0.001	—		
7. Sad	Pearson’s r	−0.378	0.184	−0.032	−0.101	−0.164	0.31	—	
	*p*-value	<0.001	0.002	0.596	0.092	0.006	<0.001	—	
8. Surprise	Pearson’s r	0.325	−0.084	−0.002	0.148	0.141	−0.129	−0.633	—
	*p*-value	<0.001	0.159	0.978	0.013	0.018	0.031	<0.001	—

**Figure A1:**
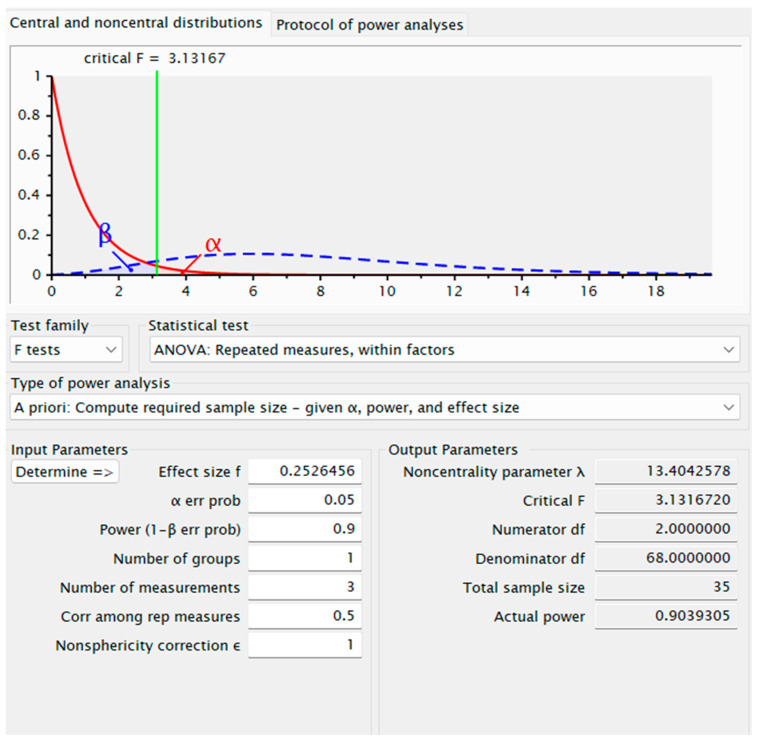
G*Power output.

**Figure A2:**
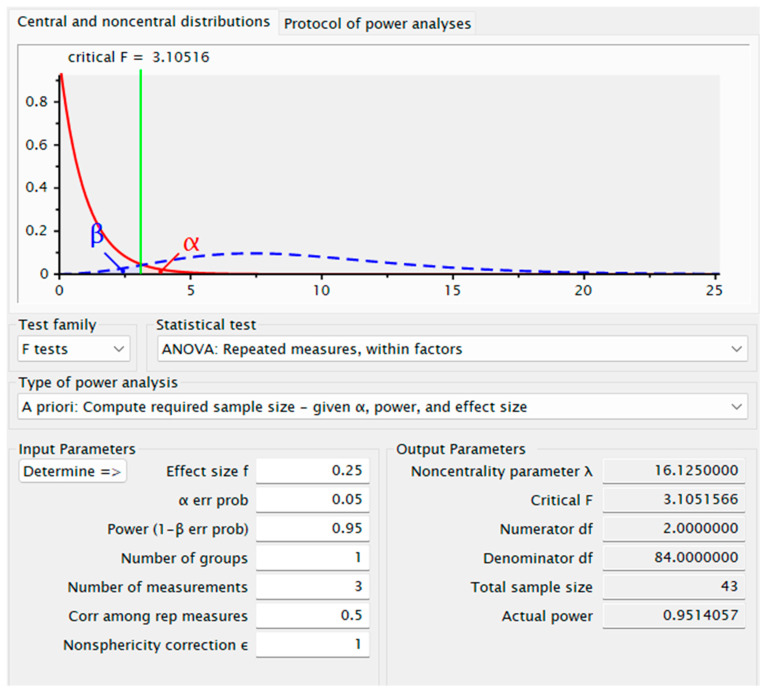
G*Power output.

**Figure A3:**
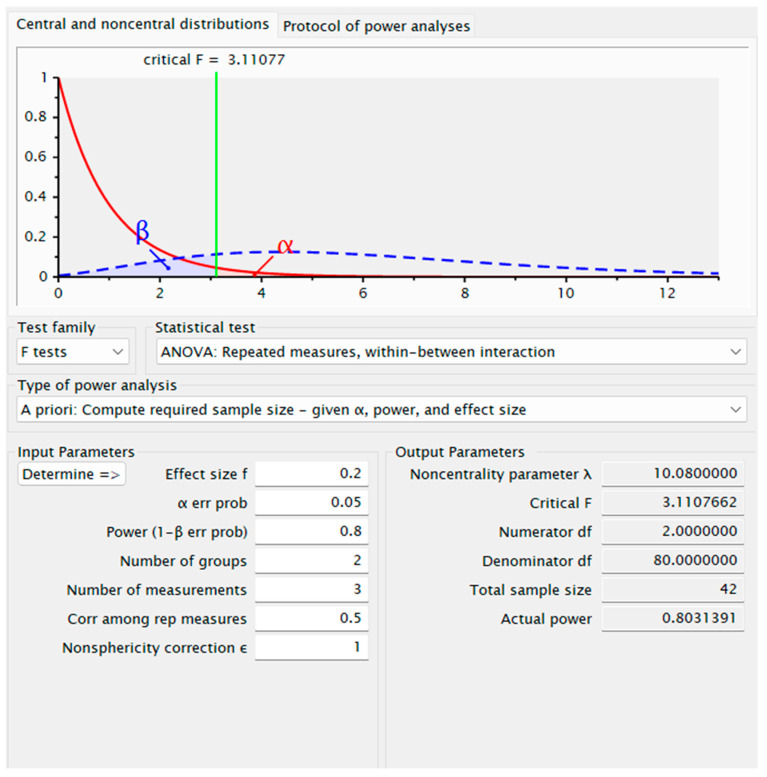
G*Power output.

## Data Availability

The data supporting this study’s findings are available in Figshare at DOI 10.6084/m9.figshare.25035098. These data were published under CC BY 4.0. Deed Attribution 4.0. International license.

## Appendix D

1. What is your general impression on the CARE webpage?
  - Very positive
  - Somewhat positive
  - Neutral
  - Somewhat negative
  - Very negative
2. How often do you visit the CARE page?
  - Never
  - Very rarely (once per month)
  - Rarely (2–3 times per month)
  - Occasionally (2–3 times per week)
  - Frequently (1–2 times per day)
  - Very frequently (more than 3 times per day)
3. How likely are you to return to the CARE webpage to search for more information and to read the latest updates?
  - Very likely
  - Somewhat likely
  - Neither likely nor unlikely
  - Somewhat unlikely
  - Very unlikely
4. What do you dislike about the CARE website?
  ______________________________________
